# Lysyl oxidase‐like 2 inhibitor rescues D‐galactose‐induced skeletal muscle fibrosis

**DOI:** 10.1111/acel.13659

**Published:** 2022-06-17

**Authors:** Yongxin Wu, Yaoxuan Wu, Yunfei Yang, Jing Yu, Jianghao Wu, Zhiyin Liao, Ai Guo, Yue Sun, Yuxing Zhao, Jinliang Chen, Qian Xiao

**Affiliations:** ^1^ Department of Geriatrics The First Affiliated Hospital of Chongqing Medical University Chongqing China

**Keywords:** aging, LOXL2, mitochondria, sarcopenia, senescence, skeletal muscle

## Abstract

Aging‐related sarcopenia is currently the most common sarcopenia. The main manifestations are skeletal muscle atrophy, replacement of muscle fibers with fat and fibrous tissue. Excessive fibrosis can impair muscle regeneration and function. Lysyl oxidase‐like 2 (LOXL2) has previously been reported to be involved in the development of various tissue fibrosis. Here, we investigated the effects of LOXL2 inhibitor on D‐galactose (D‐gal)‐induced skeletal muscle fibroblast cells and mice. Our molecular and physiological studies show that treatment with LOXL2 inhibitor can alleviate senescence, fibrosis, and increased production of reactive oxygen species in fibroblasts caused by D‐gal. These effects are related to the inhibition of the TGF‐β1/p38 MAPK pathway. Furthermore, in vivo, mice treatment with LOXL2 inhibitor reduced D‐gal‐induced skeletal muscle fibrosis, partially enhanced skeletal muscle mass and strength and reduced redox balance disorder. Taken together, these data indicate the possibility of using LOXL2 inhibitors to prevent aging‐related sarcopenia, especially with significant fibrosis.

## Graphical abstract

LOXL2 inhibitor could be promising for D‐gal‐induced skeletal muscle fibrosis and could enhance skeletal muscle mass and strength. This outcome might due to ameliorate collagen deposition caused by redox balance disorder through the TGF‐ß/p38 MAPK signaling pathway.

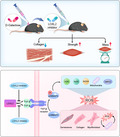

## 1 INTRODUCTION

Sarcopenia is a progressive skeletal muscle disease which is manifested as accelerated loss of muscle mass and function, with increased chance of adverse outcomes including falls, decreased function, frailty, and mortality (Cruz‐Jentoft & Sayer, 2019). The cause of sarcopenia is widely considered to be multifactorial including environmental causes, disease triggers, activation of inflammatory pathways, mitochondrial abnormalities, loss of neuromuscular junctions, reduction in the number of satellite cells, and hormonal changes (Walston, 2012). Atrophy of muscle fibers and deposition of fibrous tissue are main histopathological changes of aging‐related sarcopenia (Shang et al., 2020). Fibrous tissue plays a principal role in force transmission, maintenance and repair of muscle fibers following injury. However, excessive deposition of fibrous tissue damages muscle function and affects muscle fiber regeneration (Mahdy, 2019). Although some studies have confirmed the importance of skeletal muscle fibrosis in the pathogenesis of aging‐related sarcopenia (Wang, Wehling‐Henricks, et al., 2019), the molecular mechanisms and treatments underlying muscle fibrosis have not yet been fully elucidated.

In skeletal muscles, transforming growth factor β1 (TGF‐β1) is known as one of the most potent profibrogenic factors and regulators of fibrosis development, as it controls extracellular matrix (ECM) synthesis, remodeling, and degradation (Delaney et al., 2017). TGF‐β1 promotes fibrosis, mainly through the typical TGF‐β1/Smad signaling pathway or alternative pathways, such as the p38 mitogen‐activated protein kinase (MAPK) signaling pathway, to activate the transcription of profibrotic genes, such as Collagen‐I and Alpha‐smooth muscle actin (α‐SMA) (Sun et al., 2016). For example, myostatin, a member of the TGF‐β superfamily, induced delayed activation of p38 MAPK pathway in skeletal muscle fibroblast cells, which induced fibroblast proliferation and collagen deposition (Zhao et al., 2008). In animal experiments on the repair of rotator cuff tears, treatment with a small molecule inhibitor of p38 MAPK, SB 203580, significantly reduced the intramuscular lipid accumulation and fibrosis of the rotator cuff muscle tissue (Wilde et al., 2016). Thus, p38 MAPK pathway may be a potential therapeutic target for skeletal muscle fibrosis.

Among all the potential causes of sarcopenia, oxidative stress has emerged as a viable explanation (Foreman et al., 2021). Oxidative stress is used to describe a state caused by an imbalance between reactive oxygen species (ROS) production and scavenging (Steinz et al., 2020). In muscle fibers, ROS can be generated in varying cellular sites such as the plasma membrane, cytoplasm, and mitochondria, and are usually released in the cytoplasm (Baumann et al., 2016). Studies have found that the generation of ROS in the cell membrane and cytoplasm seems to involve membrane NADPH oxidase, and other enzymes such as xanthine oxidase (XOD) and nitric oxide synthase (NOS) have been proposed. In addition, the attenuation of antioxidant capacity also hinders the scavenging of ROS (Powers et al., 2011). Mitochondria serves as the most important cellular source of ROS (Dan Dunn et al., 2015). In aged skeletal muscle, the produce of mitochondrial ROS increased significantly, which causes mitochondrial dysfunction, altered mitochondrial dynamics, impaired mitochondrial turnover, and contributes to wasting and weakness (Boengler et al., 2017). In addition, inhibition of mitochondrial biosynthesis is accompanied by an increase in fibrosis (Dulac et al., 2020). In age‐related muscle atrophy model, regulating mitophagy to increase the mitochondrial enzyme activity and mitochondrial density maintains muscle mass and muscle strength, while reducing the content of Collagen‐I (Leduc‐Gaudet et al., 2019). ROS mediate TGF‐β1‐induced fibrosis via promoting differentiation of fibroblasts into myofibroblasts (Liu & Desai, 2015). Astaxanthin, a powerful antioxidant, has been shown to reduce ROS production in disused muscle and attenuate skeletal muscle fibrosis (Maezawa et al., 2017). The above studies indicate that ROS may be involved in the regulation of fibrosis.

Lysyl oxidase‐like 2 (LOXL2) is a copper‐dependent amine oxidase which function is to catalyze the cross‐linking of elastin and collagen in ECM (Wen et al., 2020). LOXL2 is involved in the occurrence and progression of epithelial‐mesenchymal transition, ECM deposition, and fibrosis‐related diseases (Wen et al., 2018). In mice, cardiac stress activates fibroblast cells to express and secrete LOXL2 into the interstitium, trigger fibrosis, and systolic and diastolic dysfunction of hearts (Yang et al., 2016). A study about amyotrophic lateral sclerosis (ALS) demonstrated that the expression of LOXL2 was significantly up‐regulated in gastrocnemius muscle of model group, suggesting that LOXL2 may be involved in the early neuromuscular abnormalities of ALS (de Oliveira et al., 2014). In a breast cancer study, LOXL2 induced ErbB2 activation through ROS production drives normal mammary epithelial cells to behave like cancer cells (Chang et al., 2013). In another study, inhibition of the LOXL2 expression in LM2‐4 cells resulted in strong reduction of p38 phosphorylation and inhibited their invasiveness and tumor‐forming ability (Brekhman et al., 2011). The studies indicate that ROS and p38 MAPK may be involved in the biological functions of LOXL2. Recent studies have found that selective LOXL2 inhibitors can reduce a variety of tissue fibrosis including hepatic fibrosis, cardiac interstitial fibrosis, and pulmonary fibrosis (Ikenaga et al., 2017; Jones et al., 2018; Yang et al., 2016). However, there is still a lack of research on the effect and mechanism of LOXL2 and LOXL2 inhibitors on skeletal muscle fibrosis. Herein, we investigated the effect of selective LOXL2 inhibitor on D‐galactose (D‐gal)‐induced skeletal muscle fibrosis in fibroblast cells in vitro and C57BL/6J mice in vivo, and further elucidated the underlying mechanisms.

## 2 RESULTS

### 2.1 Fibrosis was significantly increased in aged mice and D‐gal‐induced fibroblast cells

**FIGURE 1:**
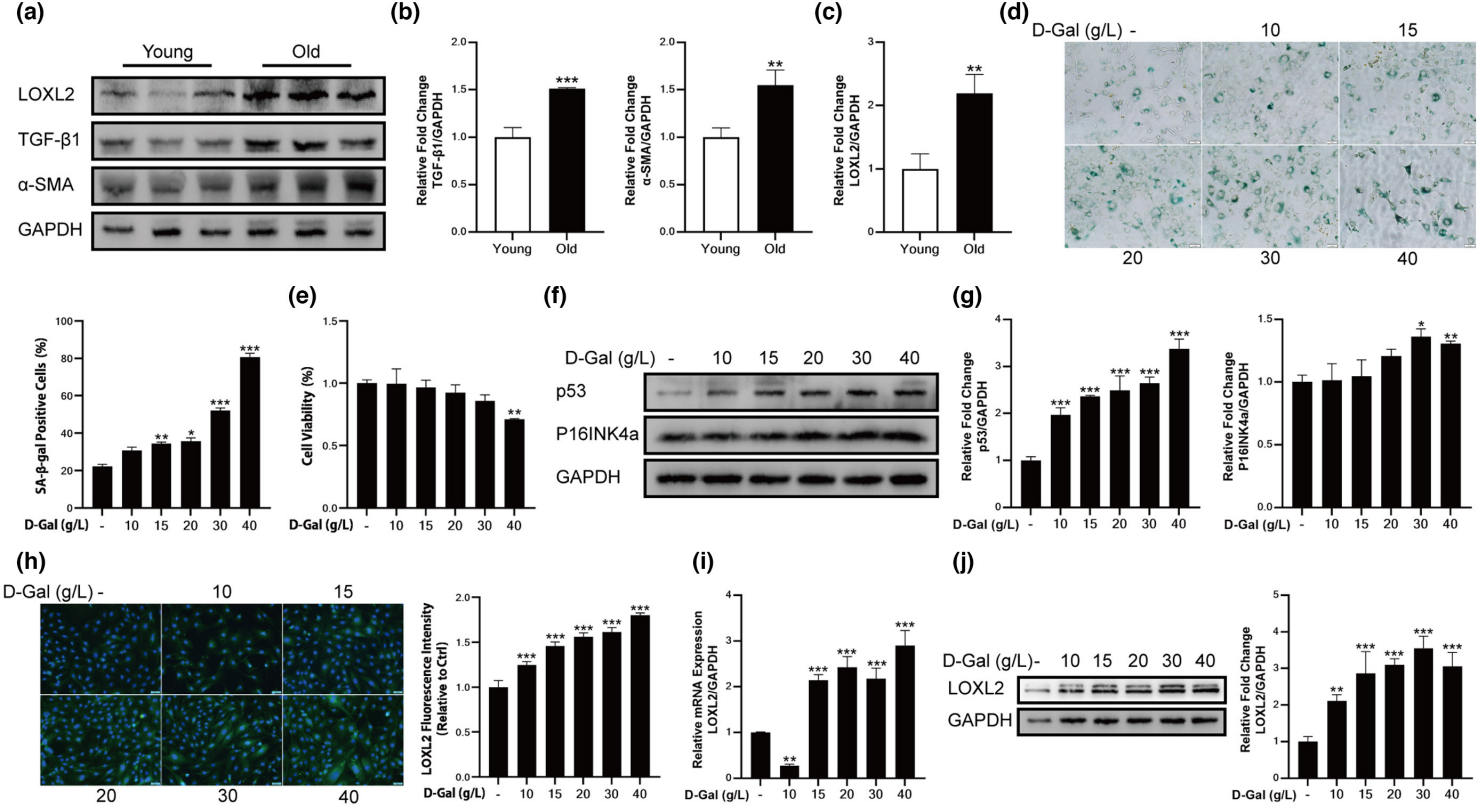
Fibrosis was increased in aged mice and D‐gal‐induced fibroblast cells (a) Detection the expression levels of LOXL2, TGF‐β1, and α‐SMA through Western blotting; GAPDH: internal reference. (b) Relative expression levels of TGF‐β1 and α‐SMA. (c) Relative expression level of LOXL2. (d) SA‐β‐gal staining of fibroblast cells. Scale bar = 50 μm. (e) Cell viability of fibroblast cells treated with different concentrations of D‐gal. (f) The expression levels of p53 and P16INK4a through Western blotting. (g) D‐gal increased p53 and P16INK4a expression dose‐dependently in fibroblast cells. GAPDH: internal reference. (h) Treatment with D‐gal increased LOXL2 expression in fibroblast cells, as assessed by immunofluorescent staining. Scale bar = 50 μm. (i) 15, 20, 30 and 40 g/L D‐gal increased LOXL2 mRNA expression level. (j) Protein expression level of LOXL2 in D‐gal treated fibroblast cells. GAPDH: internal reference. Data are shown as mean ± SD, *n* = 3. *p < 0.05, **p < 0.01, ***p < 0.001 Young vs. Old or control vs. D‐gal (10, 15, 20, 30 and 40 g/L)

We first examined the degree of skeletal muscle fibrosis in aged mice (The samples were from the previous research of our team (Lyu et al., 2019)). Western blotting results showed that compared with young group (2‐month‐old) mice, skeletal muscle fibrosis indicators including TGF‐β1 and α‐SMA were significantly increased in aged group (18‐month‐old) mice (Figure 1b). Furthermore, the expression level of LOXL2 was also significantly increased in aged mice (Figure 1c). The above results indicated that there was significant fibrosis in aged mice skeletal muscle. In the following experiments, we used D‐gal‐induced fibroblast cells and mice to explore the effect of LOXL2 inhibitor on skeletal muscle fibrosis. In this study, skeletal muscle fibroblast cells were treated with different concentrations of D‐gal (10, 15, 20, 30, 40 g/L) for 72 h. The results of senescence‐associated β‐galactosidase (SA‐beta‐gal) staining showed the number of SA‐β‐gal‐positive cells gradually increased with the increased concentration of D‐gal (Figure 1d). However, treatment with 40 g/L D‐gal showed apparent cytotoxicity on fibroblast cells compared with the control group (Figure 1e). Western blotting results indicated that P16INK4a, and p53 showed a concentration‐dependent increase (Figure 1f,g). Next, we investigated the effect of D‐gal on the expression of LOXL2 in fibroblast cells. The relative immunofluorescence intensity of LOXL2 was significantly increased in groups treated with D‐gal (Figure 1h). The result of mRNA expression showed gradient concentrations of D‐gal increased the expression level of LOXL2, compared with control group (Figure 1i). Western blotting result showed that D‐gal treatment significantly increased the expression level of LOXL2 (Figure 1j).

### 2.2 D‐gal induced fibrosis and redox balance disorder in fibroblast cells

**FIGURE 2:**
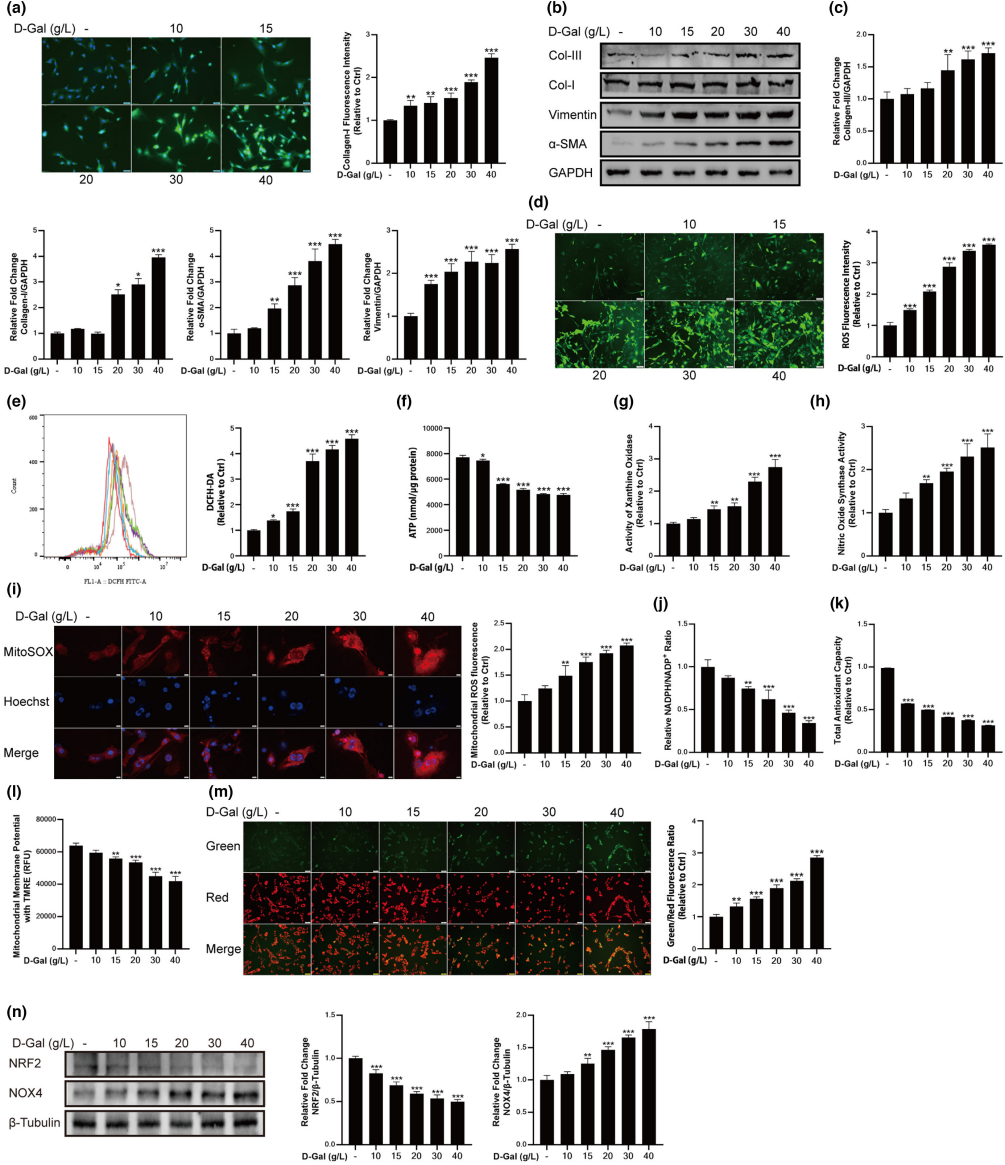
Treatment with D‐gal induced fibrosis and redox balance disorder in fibroblast cells. (a) Treatment with D‐gal increased Collagen‐I expression in fibroblast cells, as assessed by immunofluorescent staining. Scale bar = 50 μm. (b) The expression levels of fibrosis‐related proteins. (c) Treatment with D‐gal increased Collagen‐III, Collagen‐I, Vimentin and α‐SMA expression levels. GAPDH: internal reference. (d) Treatment with D‐gal increased the production of ROS, as assessed by DCFH‐DA staining. Scale bar = 100 μm. (e) Detecting the production of ROS by flow cytometry. (f) Treatment with D‐gal decreased the concentration of ATP. (g) Treatment with D‐gal increased the activity of XOD. (h) Treatment with D‐gal increased the activity of NOS. (i) Treatment with D‐gal increased the production of mitochondrial ROS, as assessed by MitoSOX Red staining. Scale bar = 20 μm. (j) The ratio of NADPH/NADP^+^. (k) The total antioxidant capacity. (l) The change of mitochondrial membrane potential was confirmed by TMRE and (m) JC‐1 probe. An increase in the ratio of green to red fluorescence intensity indicated a decrease of mitochondrial membrane potential. (n) Treatment with D‐gal decreased the expression level of NRF2 and increased the expression level of NOX4. β‐Tubulin: internal reference. Data are shown as mean ± SD, *n* = 3. *p < 0.05, **p < 0.01, ***p < 0.001 control vs. D‐gal (10, 15, 20, 30 and 40 g/L)

The immunofluorescence staining results showed that with the concentration of D‐gal increased, the expression of Collagen‐I gradually increased (Figure 2a). The results of western blotting showed that fibrosis biomarkers including Collagen‐III, Collagen‐I, Vimentin, and α‐SMA showed a dose‐dependent increase (Figure 2b,c). The findings above indicated that relatively high concentrations of D‐gal (20, 30, and 40 g/L) aggravated fibrosis in fibroblast cells. Then, we further investigated the effect of D‐gal on ROS and ATP production of fibroblast cells. In this study, the DCFH‐DA probe was used to detect intracellular ROS by fluorescence microscopy and flow cytometry. The results showed that D‐gal caused increased ROS production (Figure 2d,e). In addition, ATP production gradually decreased with the increasing concentration of D‐gal (Figure 2f). To further confirm the source of ROS, we determined XOD, NOS, and mitochondrial ROS. These enzymes and organelle are considered to be important sources of intracellular ROS. The results showed that D‐gal caused a significant increase in the activity of XOD, NOS, and the production of mitochondrial ROS (Figure 2g–i). We next examined the effects of D‐gal on the NADPH system and total antioxidant capacity, which represent important guardians in maintaining cellular redox homeostasis. Cells treated with D‐gal resulted in significantly decreased ratio of NADPH/NADP^+^ and total antioxidant capacity (Figure 2j,k). Furthermore, we examined potential changes in mitochondrial membrane potential in fibroblast cells to determine whether mitochondrial function was impaired by the elevated ROS. D‐gal treated fibroblast cells showed decreased relative fluorescence units, which indicated a decreased mitochondrial membrane potential as compared to control (Figure 2l). The disruptive effect of D‐gal on mitochondrial membrane potential was also observed visually via JC‐1 staining (Figure 2m). We also detected the factors NRF2 and NOX4 which involved in the regulation of oxidative stress by Western blotting. The results showed that with the concentration of D‐gal increased, the relative expression level of NRF2 gradually decreased (Figure 2n), while NOX4 gradually increased (Figure 2n), indicating that D‐gal caused disturbance of redox balance in fibroblast cells.

### 2.3 LOXL2 inhibitor reversed D‐gal‐induced fibrosis and redox balance disorder in fibroblast cells

**FIGURE 3:**
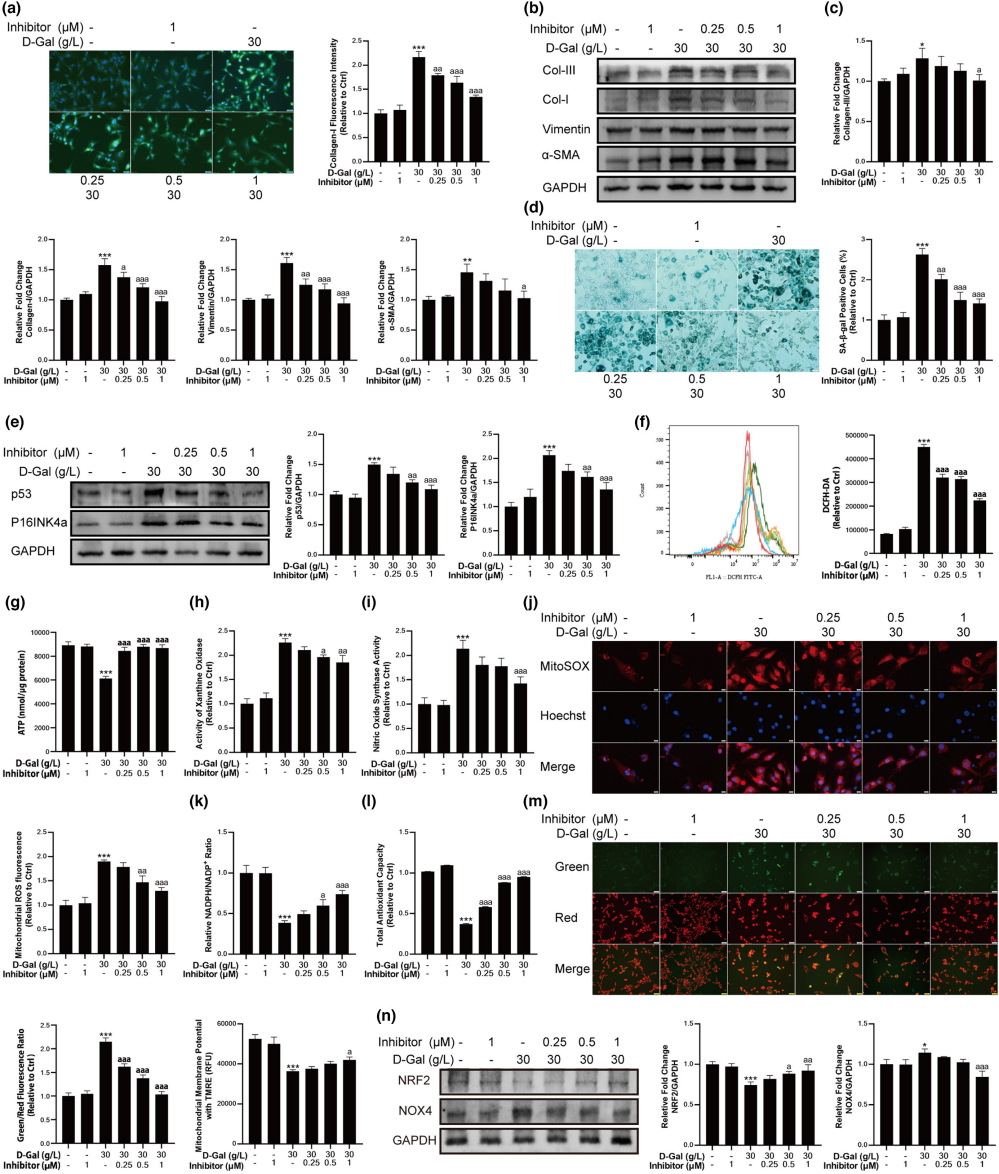
LOXL2 inhibitor reversed D‐gal‐induced fibrosis, senescence, and redox balance disorder. (a) LOXL2 inhibitor decreased the content of Collagen‐I in D‐gal‐treated fibroblast cells, as assessed by immunofluorescent staining. Scale bar = 50 μm. (b) The expression levels of fibrosis‐related proteins. (c) LOXL2 inhibitor decreased the expression levels of Collagen‐III, Collagen‐I, Vimentin, and α‐SMA. GAPDH: internal reference. (d) LOXL2 inhibitor reduced the number of SA‐β‐gal‐positive cells in D‐gal‐treated fibroblast cells. Scale bar = 50 μm. (e) LOXL2 inhibitor decreased the expression levels of p53 and P16INK4a. GAPDH: internal reference. (f) LOXL2 inhibitor reduced the production of ROS. (g) LOXL2 inhibitor increased the concentration of ATP in D‐gal‐treated fibroblast cells. (h) 0.5 and 1 μm LOXL2 inhibitors decreased the activity of XOD in D‐gal‐treated fibroblast cells. (i) 1 μm LOXL2 inhibitor decreased the activity of NOS in D‐gal‐treated fibroblast cells. (j) LOXL2 inhibitors decreased the production of mitochondrial ROS, as assessed by MitoSOX Red staining. Scale bar = 20 μm. (k) LOXL2 inhibitors increased the ratio of NADPH/NADP^+^ and (l) total antioxidant capacity in D‐gal‐treated fibroblast cells. (m) The change of mitochondrial membrane potential was confirmed by TMRE and JC‐1 probe. An increase in the ratio of green to red fluorescence intensity indicated a decrease of mitochondrial membrane potential. (n) Treatment with LOXL2 inhibitor increased the expression level of NRF2 and decreased the expression level of NOX4. GAPDH: internal reference. Data are shown as mean ± SD, *n* = 3. *p < 0.05, **p < 0.01, ***p < 0.001 control vs. 30 g/L D‐gal. ^a^ *p* < 0.05, ^aa^ *p* < 0.01, ^aaa^ *p* < 0.001 30 g/L D‐gal vs. LOXL2 inhibitor (0.25, 0.5 and 1 μM)

According to the above findings, in subsequent experiments, we used 30 g/L D‐gal to induce cell fibrosis. By applying a gradient of LOXL2 inhibitors to D‐gal‐treated fibroblast cells, we found that the fluorescence intensity of Collagen‐I decreased with the increased concentration of LOXL2 inhibitors (Figure 3a). The results of Western blotting showed that after treatment with LOXL2 inhibitor, the expression level of fibrosis‐related proteins decreased in D‐gal‐induced cells (Figure 3b,c). The results of SA‐β‐gal staining showed that treatment with LOXL2 inhibitor reduced the number of SA‐β‐gal‐positive cells (Figure 3d), and the expression of senescence‐related proteins including p53 and P16INK4a also decreased (Figure 3e). Furthermore, the production of ROS decreased, and the production of ATP increased after LOXL2 inhibitors treatment (Figure 3f,g). By further confirming the source of ROS, we found that LOXL2 inhibitors reduced the activities of XOD and NOS and suppressed mitochondrial ROS production in D‐gal‐treated fibroblast cells (Figure 3h–j). In addition, compared with D‐gal‐treated cells, the ratio of NADPH/NADP^+^ and total antioxidant capacity were also increased, confirming that LOXL2 inhibitors enhanced intracellular antioxidant capacity (Figure 3k,l). Next, we determined the changes in mitochondrial membrane potential after the reduction of ROS production, the results showed that the mitochondrial membrane potential gradually increased with the increasing concentration of LOXL2 inhibitor, indicating that mitochondrial function was partially restored (Figure 3m). Finally, we validated the changes in NRF2 and NOX4 expression levels. LOXL2 inhibitors increased the protein expression level of NRF2 and decreased the expression level of NOX4 (Figure 3n), which indicated that LOXL2 inhibitor alleviated D‐gal‐induced oxidative stress in fibroblast cells.

### 2.4 LOX2 inhibitor reversed fibrosis via TGF‐β1‐mediated p38 MAPK signaling pathway

**FIGURE 4:**
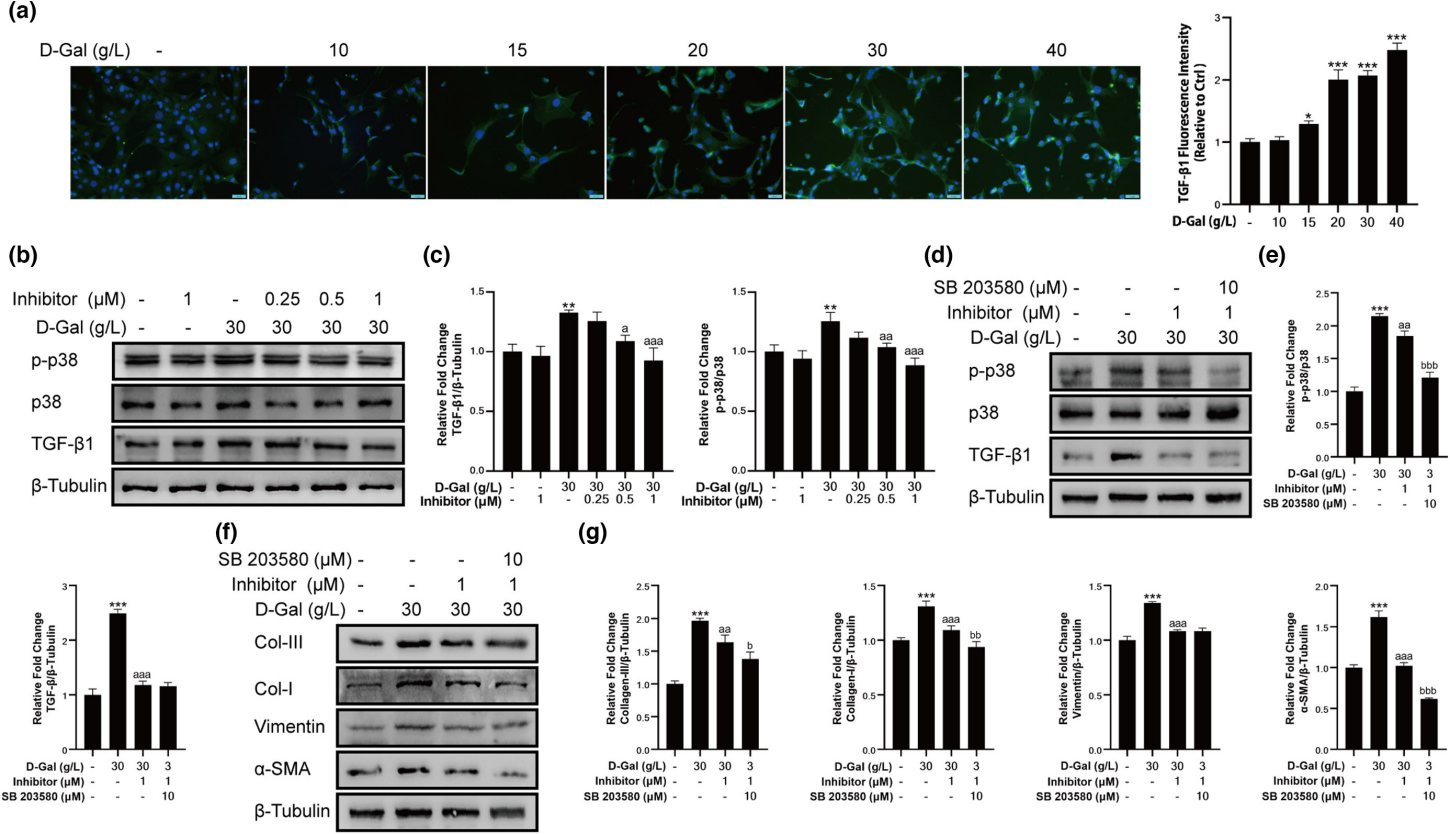
LOXL2 regulated the TGF‐β1‐mediated p38 MAPK signaling pathway. (a) D‐gal increased the content of TGF‐β1 in fibroblast cells, as assessed by immunofluorescent staining. Scale bar = 50 μm. (b) The expression levels of TGF‐β1, p‐p38, and p38. (c) LOXL2 inhibitor decreased the protein expression levels of p‐p38 and TGF‐β1 in D‐gal treated fibroblast cells. β‐Tubulin: internal reference. (d) The expression levels of TGF‐β1, p‐p38, and p38. (e) The protein expression levels of p‐p38 and TGF‐β1. β‐Tubulin: internal reference. (f) The expression levels of fibrosis‐related proteins. (g) SB 203580 further reduced the protein expression levels of Collagen‐III, Collagen‐I, and α‐SMA in D‐gal together with LOXL2 inhibitor‐treated fibroblast cells. Data are shown as mean ± SD, *n* = 3. *p < 0.05, **p < 0.01, ***p < 0.001 control vs. 30 g/L D‐gal. ^a^ *p* < 0.05, ^aa^ *p* < 0.01, ^aaa^ *p* < 0.001 30 g/L D‐gal vs. LOXL2 inhibitor (0.25, 0.5 and 1 μM) or 30 g/L D‐gal vs. LOXL2 inhibitor. SB 203580. ^b^ *p* < 0.05, ^bb^ *p* < 0.01, ^bbb^ *p* < 0.001 SB 203580 vs. LOXL2 inhibitor

TGF‐β1 is an important fibrogenic factor in skeletal muscle. We found that the expression level of TGF‐β1 in fibroblast cells increased with the increased concentration of D‐gal (Figure 4a). However, treatment with LOXL2 inhibitor decreased the expression level of TGF‐β1 in D‐gal‐induced cells (Figure 4b,c). p38 MAPK is an important downstream molecule of TGF‐β1. We found that p‐p38 also showed a dose‐dependent decrease with the treatment of LOXL2 inhibitors (Figure 4c). Furthermore, we applied SB 203580 to fibroblast cells treated with D‐gal and LOXL2 inhibitor. The results showed that as the expression level of p‐p38 decreased (Figure 4d,e), fibrosis indicators including Collagen‐III, Collagen‐I, and α‐SMA showed a further decrease (Figure 4f,g). The results showed that LOXL2 inhibitor reversed D‐gal‐induced fibrosis by down‐regulating the TGF‐β1‐mediated p38 MAPK signaling pathway.

### 2.5 LOXL2 inhibitor reversed D‐gal‐induced senescence and skeletal muscle fibrosis in mice

**FIGURE 5:**
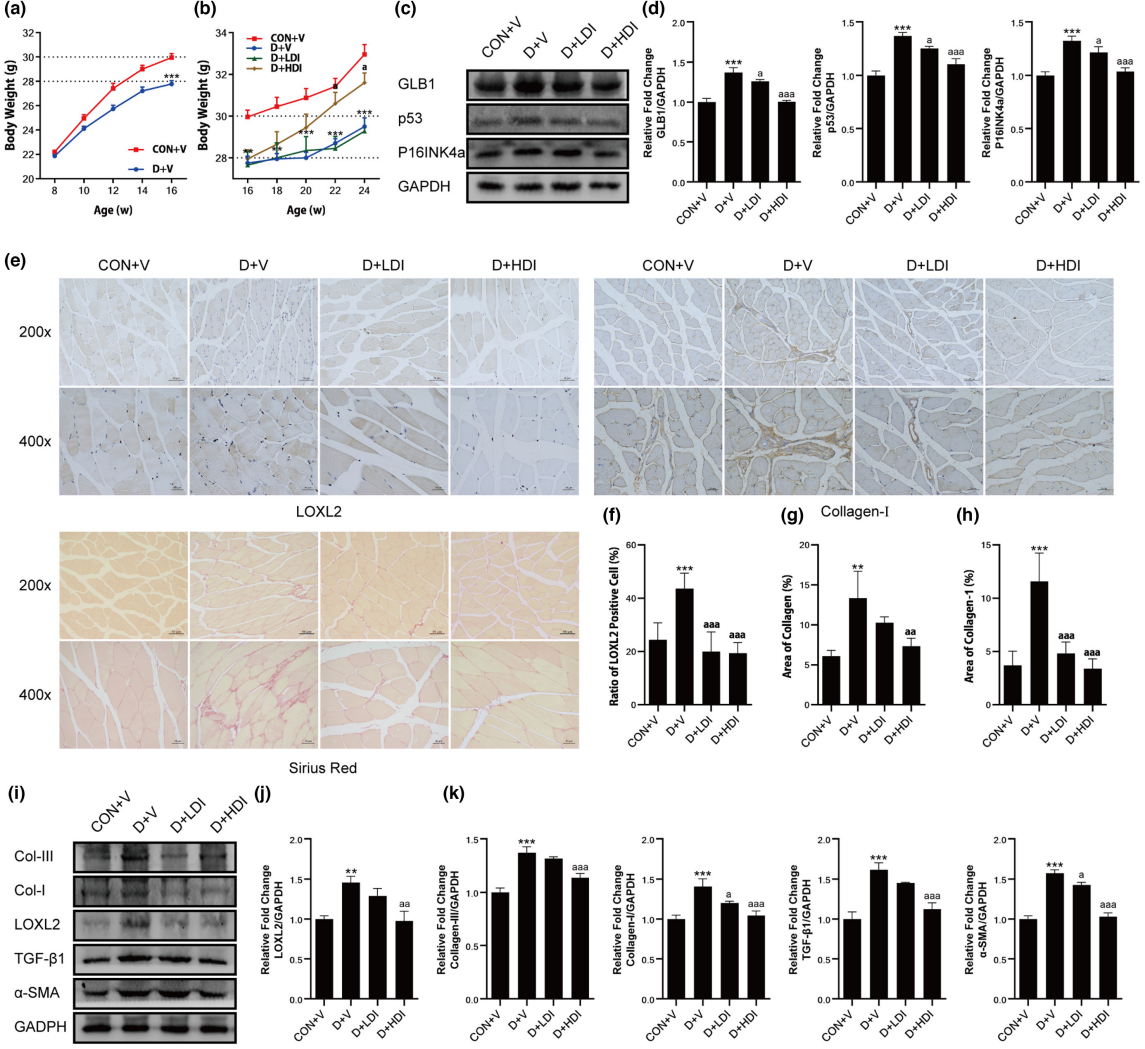
LOXL2 inhibitor protected against D‐gal‐induced senescence and skeletal muscle fibrosis in mice. (a) Body weight from 8 to 16 weeks old. (b) Body weight from 16–24 weeks old. (c) The expression levels of GLB1, p53, and P16INK4a. (d) LOXL2 inhibitor decreased the expression levels of GLB1, p53, and P16INK4a in D‐gal‐treated mice. GAPDH: internal reference. (e) The immunohistochemical staining of LOXL2 and Collagen‐I and Sirius Red staining. Scale bar = 20 or 50 μm. (f) LOXL2 inhibitor reduced the content of LOXL2. (g) Area of collagen in mice with different treatment. (h) LOXL2 inhibitor reduced the content of Collagen‐I. (i) The expression levels of LOXL2 and fibrosis‐related proteins. (j) High‐dose LOXL2 inhibitor reduced the expression of LOXL2. GAPDH: internal reference. (k) High‐dose LOXL2 inhibitor reduced the expression of Collagen‐III, Collagen‐I, TGF‐β1 and α‐SMA. GAPDH: internal reference. Data are shown as mean ± SD or SEM, *n* = 9–11 or 3. **p < 0.01, ***p < 0.001 C + V vs. D + V. ^a^ *p* < 0.05, ^aa^ *p* < 0.01, ^aaa^ *p* < 0.001 D + V vs. D + LDI or D + HDI

To further study the effect of LOXL2 inhibitor on skeletal muscle fibrosis, we induced senescence in mice by intraperitoneal injection of D‐gal and used them for subsequent experiments. We injected 200 mg/kg D‐gal into the intraperitoneal cavity of 8‐week‐old C57BL/6J mice daily for 8 weeks. After 8 weeks of treatment, the body weight of mice in D‐gal groups (D + V group) was significantly lower than that in control group (CON + V group) (Figure 5a), indicating that D‐gal treatment caused metabolic disorders in mice. Next, while injecting D‐gal, the D + LDI and D + HDI groups were intraperitoneally injected with different concentrations of LOXL2 inhibitors (D + LDI group: 5 mg/kg; D + HDI group: 10 mg/kg) daily for 8 weeks at the same time. The results of body weight showed that 8 weeks of high‐dose LOXL2 inhibitor treatment increased the body weight of mice, compared with D + V group (Figure 5b). The results indicated that high‐dose LOXL2 inhibitor alleviated the metabolic disorder caused by D‐gal. Further, we examined the expression levels of senescence‐related proteins in each group of mice. The results showed that, compared with CON + V group, the expression level of GLB1, p53, and P16INK4a increased in D + V group. While treatment with LOXL2 inhibitors, no matter low‐ or high‐dose inhibitor decreased the expression levels (Figure 5c,d), which showed that treatment with LOXL2 inhibitor alleviated D‐gal‐induced senescence in mice. Next, we performed immunohistochemical staining of LOXL2 in gastrocnemius muscle of each group. Compared with CON + V group, the expression level of LOXL2 in D + V group increased, while the expression levels in D + LDI group and D + HDI group were significantly decreased (Figure 5f). Western blotting results further confirmed that the expression level of LOXL2 in D + V group was higher than in CON+V group. In addition, the expression levels of D + LDI and D + HDI groups were significantly reduced (Figure 5j). These results indicated that LOXL2 inhibitor significantly reduced the amount of LOXL2. Next, we detected the collagen area of gastrocnemius in each group. The results of Sirius red staining and Collagen‐I immunohistochemical staining showed that the area of collagen deposition in D + V group significantly increased (Figure 5g,h). Sirius red staining revealed high‐dose inhibitor significantly decreased the area of collagen (Figure 5g); immunohistochemical staining further showed both high‐ and low‐dose inhibitors reduced the expression of Collagen‐I (Figure 5h). Further examining the fibrosis‐related proteins in gastrocnemius of each group, we found that compared with CON + V group, the expression level of Collagen‐III, Collagen‐I, TGF‐β1, and α‐SMA in D + V group was significantly increased, while decreased in both D + LDI group and D + HDI group, although some of the data lacked statistical significance (Figure 5k). These results showed that LOXL2 inhibitor significantly reduced fibrosis in gastrocnemius muscle of mice.

### 2.6 LOXL2 inhibitor ameliorated D‐gal‐induced skeletal muscle atrophy and redox balance disorder

**FIGURE 6:**
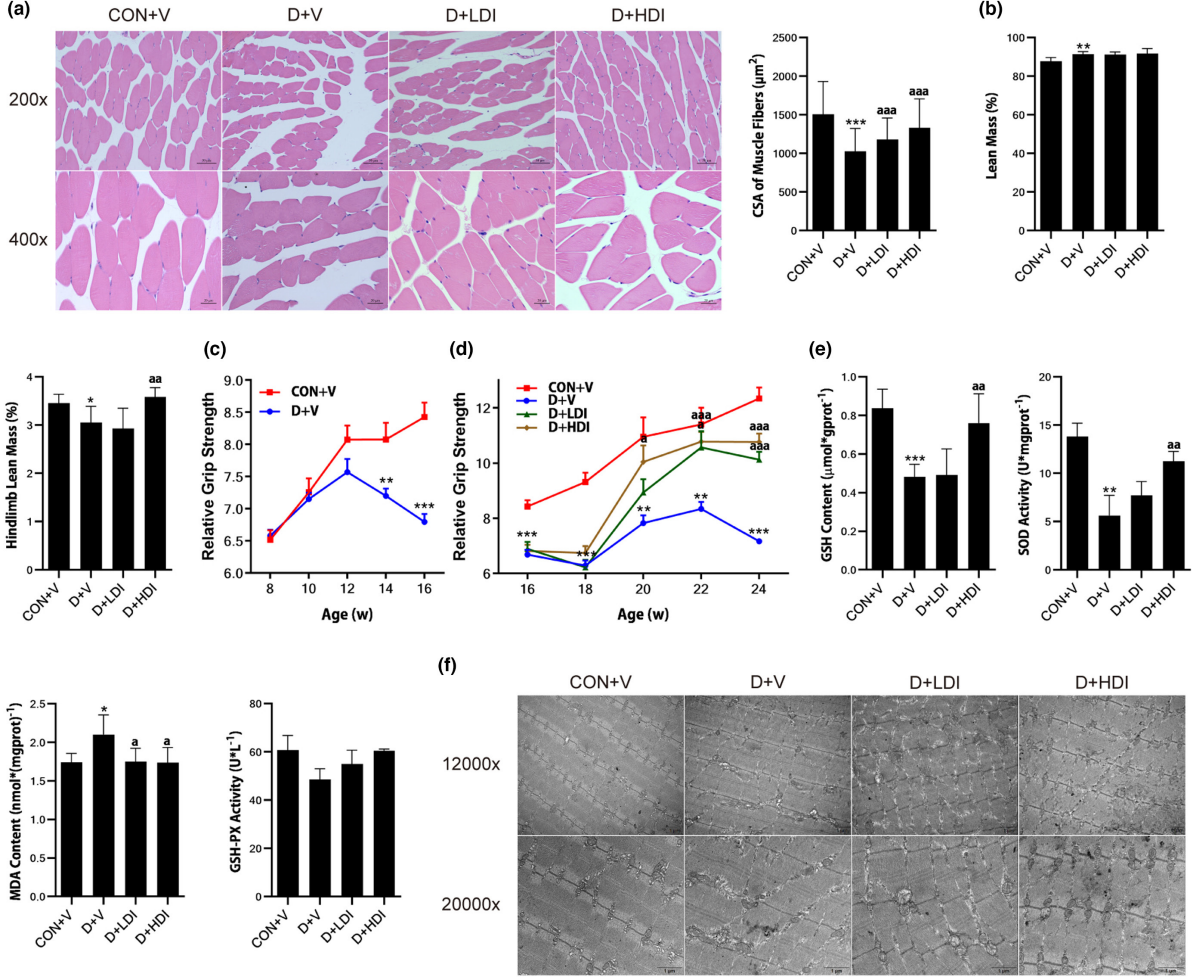
LOXL2 inhibitor ameliorated D‐gal‐induced skeletal muscle atrophy and redox balance disorder. (a) CSA of muscle fibers (μm^2^) were assessed by H&E staining. Scale bar = 20 or 50 μm. (b) Lean mass and hindlimb lean mass. (c) Relative grip strength form 8–16 weeks old. (d) Relative grip strength form 16–24 weeks old. (e) Changes of oxidative stress biomarkers in mice with different treatment. (f) The electron microscope images of mitochondria with 12,000 or 20,000× objective. Scale bar = 1 μm. Data are shown as mean ± SD or SEM, *n* = 9–11 or 3. *p < 0.05, **p < 0.01, ***p < 0.001 C + V vs. D + V. ^a^ *p* < 0.05, ^aa^ *p* < 0.01, ^aaa^ *p* < 0.001 D + V vs. D + LDI or D + HDI

Although the function of LOXL2 is to catalyze the cross‐linking of elastin and collagen, skeletal muscle as a whole, the reduction of fibrosis may have an impact on the recovery of muscle mass and function. Therefore, we further investigated the effect of LOXL2 inhibitor on the mass and function of skeletal muscle. The results of H&E staining of gastrocnemius muscle showed that in D + V group, the cross‐sectional area (CSA) of muscle fibers was significantly decreased. While both low‐ and high‐dose inhibitors significantly increased the CSA (Figure 6a), they were still lower than that in CON + V group. The dual‐energy X‐ray absorptiometry (DEXA) test indicated that mice in D + V group showed a significant decrease in hindlimb lean mass (Figure 6b). However, compared with D + V group, the hindlimb lean mass of mice in D + HDI group was increased significantly (Figure 6b). In addition to the increased hindlimb lean mass, we found that from the sixth week of treatment (14‐week‐old mice), the relative grip strength of D‐gal‐induced mice began to decrease significantly (Figure 6c). At the same time, starting from the twelfth week (20‐week‐old), the relative grip strength of mice treated with LOXL2 inhibitors gradually increased (Figure 6d). Normally, the function of muscle is closely related to the state of skeletal muscle redox balance. Therefore, we used corresponding assay kit to detect the content or activity of oxidative stress biomarkers in muscle samples of each group. The results showed that in D + V group, the activity of SOD, the content of GSH decreased and the content of MDA increased, while high‐dose LOXL2 inhibitor significantly reversed the above changes (Figure 6e). The above findings indicated that the LOXL2 inhibitor exhibited a significant antioxidant effect. We further detected the integrity of gastrocnemius mitochondria in each group using scanning electron microscopy. Electron microscope images showed that in gastrocnemius muscle of mice injected with D‐gal, the arrangement of myofilaments was disordered and the sarcomere was blurred, some mitochondria were swollen, the arrangement of mitochondrial cristae was disordered, and a few mitochondrial membranes were ruptured and the contents flowed out, while LOXL2 inhibitors, especially high‐dose inhibitor, partially attenuated the abnormalities of D‐gal‐induced myofiber structure and mitochondrial damage (Figure 6f). The above results suggested that LOXL2 inhibitor may promote the recovery of skeletal muscle mass and function by promoting the restoration of redox balance and producing lower ROS to prevent further mitochondrial damage.

## 3 DISCUSSION

In this study, we found that the presence of skeletal muscle fibrosis in aged mice may be related to the high expression of LOXL2 in skeletal muscles. In vitro, D‐gal induced mitochondrial dysfunction and alterations in p38 MAPK signaling pathway in fibroblast cells. Treatment with LOXL2 inhibitor can reduce ROS production, protect mitochondrial structure, and decrease the activation of p38, thereby reducing fibrosis. In vivo, LOXL2 inhibitor can reduce fibrosis and redox balance disorder in D‐gal‐induced aging mice, alleviate skeletal muscle atrophy and enhance the strength of skeletal muscles.

According to the 2018 operational definition of sarcopenia by EWGSOP2, low muscle strength identifies probable sarcopenia and low muscle quantity or quality confirms the diagnose of sarcopenia (Cruz‐Jentoft et al., 2019). Low muscle quantity or quality is manifested by replacement of muscle fibers with fat and increased fibrous tissue (Dhillon & Hasni, 2017). In this study, we found that compared with young mice, the expression levels of TGF‐β1 and α‐SMA were significantly increased in aged mice, which agreed with previous study indicating that aged mice showed obvious fibrosis in skeletal muscles (Shang et al., 2020). In addition, the increase of LOXL2 in skeletal muscle indicated that LOXL2 may be involved in aging‐related skeletal muscle fibrosis. On the one hand, fibrous tissue involved in the repair of muscle fibers, and on the other hand, excessively deposited fibrous tissue damaged muscle function (Mahdy, 2019). Therefore, the treatment of skeletal muscle fibrosis may contribute to the treatment of sarcopenia.

D‐galactose, as an important compound for investigating aging‐related diseases, has been applicated in a variety of aging disease models (Azman & Zakaria, 2019; Xu et al., 2020). Moreover, D‐gal can cause fibrosis in many tissues including skeletal muscles (Miao et al., 2019). Therefore, in this study, both in vivo and in vitro experiments, we used D‐gal to induce aging‐related skeletal muscle fibrosis. In vitro experiments, we found that D‐gal induced fibrosis in skeletal muscle fibroblast cells and increased expression of LOXL2, indicating that LOXL2 is involved in fibrosis caused by D‐gal. Our research further found that D‐gal also caused increased production of ROS and mitochondrial dysfunction in fibroblasts, indicating that mitochondria may be involved in the process of fibrosis, which is consistent with previous studies that D‐gal caused mitochondrial damage (Guo et al., 2020; Zhou et al., 2019). In vivo experiments, we found that D‐gal caused weight loss, reduced grip strength, reduced lean mass, and hindlimb lean mass, indicating that D‐gal induced the symptoms and manifestations of sarcopenia in mice. In addition, we found that the CSA of skeletal muscle fibers decreased, the area of fibrosis increased, the oxidative stress indicators were abnormal and structure of mitochondria changed. These findings indicated that D‐gal induced skeletal muscle atrophy, skeletal muscle fibrosis, and redox balance disorder in mice.

Inhibiting aging‐related skeletal muscle fibrosis contributes to the recovery of skeletal muscle mass and function. In vitro experiments, after treatment with LOXL2 inhibitor, we found that the expression of fibrosis‐related proteins decreased in D‐gal‐induced fibroblasts, the production of ROS and the mitochondrial dysfunction were reduced. In vivo experiments, treatment with LOXL2 inhibitor reduced the area of fibrosis and collagen deposition. Moreover, oxidative stress was inhibited, D‐gal‐induced myofilament arrangement disorder, sarcomere blurring, partial mitochondrial swelling, and mitochondrial cristae arrangement disorder were alleviated. Together with skeletal muscle mass and grip strength was restored. The results showed that in addition to effectively reducing fibrosis, LOXL2 inhibitor may also promote the recovery of skeletal muscle function by alleviating redox balance disorder and protecting the structure of mitochondria through lower ROS production, although the mechanism is still unclear. A study suggested that the LOX family may be a new source of ROS, as the LOX family produces H_2_O_2_ as a by‐product while catalyzing the cross‐linking of collagen and elastin (Varona et al., 2017). After examining the blood vessel wall of LOX transgenic mice, it was found that the content of H_2_O_2_ and O^2−^ in the vessel wall was increased, with the activity of NADPH oxidase enhanced and mitochondrial membrane potential decreased (Martínez‐Revelles et al., 2017). Another study found that β‐aminopropionitrile, an inhibitor of LOX activity, can reduce ROS production in heart and blood vessel cells (Martínez‐Martínez et al., 2016). Combined with the results of this study, LOXL2 may be involved in the production of ROS and other enzymes and proteins related to ROS production, thereby affecting mitochondrial function, and then participate in the metabolism and function of cells, tissues, and organs.

TGF‐β1 plays an important role in muscle impairment and fibrosis that accompanies the aging process (Ismaeel et al., 2019). During normal aging, muscle cells increase the level of TGF‐β1 and change to a more fibrotic phenotype (Mann et al., 2011). The pathways of TGF‐β1 involved in skeletal muscle fibrosis mainly include the classic TGF‐β1/Smad pathway and the TGF‐β1/p38 MAPK alternative pathway (Ismaeel et al., 2019). A study found that in the process of using ganoderic acid to treat renal fibrosis, ganoderic acid inhibited the progression of renal fibrosis by suppressing the activation of p38 (Geng et al., 2020). In addition, it was discovered that DR8, a polypeptide with strong antioxidant activity, can prevent pulmonary fibrosis by inhibiting the TGF‐β1/p38 MAPK pathway (Wang, Yan, et al., 2019). In this study, we found that the expression levels of TGF‐β1 and p‐p38 in D‐gal‐induced fibroblasts were up‐regulated, indicating that the TGF‐β1/p38 MAPK signaling pathway was activated in fibroblasts. After treatment with LOXL2 inhibitor, the expression of p‐p38 decreased and inhibited the expression of fibrosis genes, indicating that during the process of skeletal muscle fibrosis, LOXL2 may promote p38 phosphorylation to activate the TGF‐β1/p38 MAPK signaling pathway, while LOXL2 inhibitor can inhibit the TGF‐β1/p38 MAPK signaling pathway to prevent and treat fibrosis.

In summary, our results indicate that in vitro, LOXL2 inhibitor can reduce D‐gal‐induced fibroblast fibrosis by inhibiting TGF‐β1/p38 MAPK activation and alleviating redox balance disorder. In vivo, LOXL2 inhibitor can reduce D‐gal‐induced skeletal muscle fibrosis, inhibit oxidative stress, promote the increase of skeletal muscle mass and strength which plays a protective role in sarcopenia. Although our study on the process of fibrosis is still relatively superficial, our findings provide new ideas for the treatment of sarcopenia.

## 4 METHODS

### 4.1 Reagents and antibodies

Selective LOXL2 inhibitor ([2‐Chloropyridin‐4‐yl] methanamine hydrochloride) and selective p38 MAPK inhibitor (SB 203580) were purchased from MedChemExpress. The inhibitors were dissolved in DMSO. Phosphate buffered saline (PBS) powder, Dulbecco's modified eagle medium (DMEM), penicillin–streptomycin (P/S, 10,000 U/ml of penicillin and 10,000 μg/ml of streptomycin), fetal bovine serum, and pancreatin with 0.25% EDTA were purchased from Shanghai Zhong Qiao Xin Zhou biotechnology. D‐gal was obtained from Sigma‐Aldrich. All primary and secondary antibodies were purchased from Abcam, Proteintech, Affinity Biosciences.

### 4.2 Skeletal muscle fibroblast cell culture

Mouse skeletal muscle fibroblast cells were obtained from Shanghai Zhong Qiao Xin Zhou biotechnology and maintained in DMEM with 20% fetal bovine serum and 1% P/S. To initiate fibrosis, the cells were grown to 70–80% confluence and incubated in DMEM containing 20% fetal bovine serum and 1% P/S together with different concentrations of D‐gal for 72 h in order to obtain the most suitable concentration for fibrosis. In the following experiments, the fibroblast cells were treated with D‐gal, with or without different concentrations of LOXL2 inhibitor and SB 203580 for 72 h. DMSO was used as a vehicle control. The final concentration of DMSO was <0.1%.

### 4.3 Cell viability

Cell viability was determined by cell counting kit‐8 assay (CCK‐8, Dojindo Laboratories). Fibroblast cells were seeded in 96‐well plates at a density of 1 × 10^5^ cells per well. After cultured in complete medium for 24 h, the fibroblasts were treated with different concentrations of D‐gal for 72 h. Then, cell viability was determined by incubation with DMEM containing CCK‐8 for 40 min. The absorbance at 450 nm was measured by microplate reader.

### 4.4 Western blot analysis

Fibroblast cells or skeletal muscle samples were lysed with cold RIPA buffer containing with PMSF on ice for 30 min. Lysates were centrifuged at 12,000 *g* for 15 min, and the supernatants were transferred into new tubes. Protein concentration of each sample was quantified using a BCA protein assay kit (Beyotime Biotechnology). The same amounts of proteins (30 μg) were separated by 8%, 10%, or 12% SDS‐PAGE, transferred to PVDF membranes, blocked with QuickBlockTM Western blocking solution (Beyotime Biotechnology) for 30 min at room temperature, and incubated with primary antibody for 14 h at 4°C. After washing with TBST three times, a secondary antibody was added and incubated for 2 h at room temperature. Washing again with TBST three times. Signals were detected using an enhanced chemiluminescence substrate (Zenbio). Then, specific protein bands were visualized using the Fusion FX.EDGE Imaging System. Intensity of individual bands in Western blots was quantitated using Evolution‐Capt software. The measure of protein relative abundance in the different samples was expressed relative to reference protein signal. The relative abundance of D‐gal‐treated, LOXL2 inhibitor‐treated and SB 203580‐treated groups was normalized by that of control group.

### 4.5 SA‐β‐gal staining

SA‐β‐gal staining was performed using a SA‐β‐gal staining kit (Beyotime Biotechnology). Fibroblast cells were seeded in six‐well plates. The first experiment involved control group and different concentrations of D‐gal groups. The second experiment involved control group, D‐gal group, and different concentrations of LOXL2 inhibitor groups. Then, cells in all groups were stained using the SA‐β‐gal staining kit and cultured in a 37°C dry incubator (without CO_2_). The SA‐β‐gal‐positive cells exhibited in blue color. The number of positive cells was counted under a phase‐contrast microscope.

### 4.6 Quantitative real‐time polymerase chain reaction (qRT‐PCR)

All gene primers were synthesized in Takara bio and shown in Table 1. According to the instructions of manufacturer, the total RNA was extracted using TRIzol total RNA extraction. RNA concentration and purity were measured using a spectrophotometer (NanoDrop® ND‐2000, Thermo scientific). According to the protocol of manufacturer, reverse‐transcribed RNA samples to obtain cDNA. qRT‐PCR was performed with cDNA, SYBR Green PCR Master Mix, and primers. The data were analyzed by the 2‐ΔΔCt threshold cycle method.

**TABLE 1:** Primers used for the qRT‐PCR analysis

Gene name	Sequence 5′‐3′ (Forward)	Sequence 5′‐3′ (Reverse)
LOXL2	ACCCCAACTATGAAGTGCCAG	TGGGGTTGATACTTCACGGTC
GAPDH	AATGGATTTGGACGCATTGGT	TTTGCACTGGTACGTGTTGAT

### 4.7 Intracellular ROS level

The intracellular ROS levels were measured using a ROS Assay Kit (Beyotime Biotechnology). After indicated treatment, cells cultured in six‐well plates were incubated with 2′, 7′‐dichlorofluorescein‐diacetate (DCFH‐DA) diluted in serum‐free medium at 37°C for 20 min. Capture the images and use Image‐J software to measure the fluorescence intensity. The cells used for flow cytometry were resuspended in DMEM with DCFH‐DA and incubated at 37°C for 20 min. Finally, measured samples by flow cytometry.

### 4.8 Intracellular relative ATP content

The intracellular ATP content was measured using an ATP content kit (Beyotime Biotechnology). Cells in six‐well plates after indicated treatment were lysed with lysate buffer. Lysates were centrifuged at 12,000 *g* for 5 min. Protein concentration of each sample was quantified using a BCA protein assay kit. Samples were added into ATP detection solution, and the ATP contents were measured with a fluorescence microplate reader. The relative ATP content is further standardized by the protein content.

### 4.9 XOD activity

The XOD activity was measured using a Xanthine oxidase activity Assay Kit (Makclin Biochemical). Cells in six‐well plates after indicated treatment were collected into extract, sonicated and centrifuged at 8000 *g* for 10 min. The supernatant was collected for detection. XOD working solution was added to the supernatant, mixed immediately, and recorded the initial absorbance and the absorbance after 1 min at 290 nm using a microplate reader. The XOD activity was further standardized by the protein concentration.

### 4.10 NOS activity

The activity of NOS was measured using Nitric Oxide Synthase Assay Kit (Beyotime Biotechnology). After indicated treatment, cells cultured in 96‐well plates were incubated with NOS assay buffer for 30 min at 37°C. Fluorescence microplate reader was used to detect the fluorescence intensity of each well (Ex/Em: 495/515 nm).

### 4.11 Detection of mitochondrial ROS, mitochondrial membrane potential

#### 4.11.1 Mitochondrial ROS

The mitochondrial ROS was detected using MitoSOX™ Red mitochondrial superoxide indicator (Invitrogen). After indicated treatment, cells cultured in 24‐well plates were incubated with 5 μM MitoSOX™ Red superoxide indicator for 30 min at 37°C. Cells were then washed with PBS for three times. Capture the images using fluorescence microscope and use Image‐J software to measure the fluorescence intensity.

#### 4.11.2 Mitochondrial membrane potential with JC‐1 and TMRE

Mitochondrial membrane potential in fibroblast cells were measured using JC‐1 probe (Solarbio) or TMRE probe (Beyotime Biotechnology). Cells cultured in 96‐well plates after indicated treatment were incubated with 100 μl JC‐1 staining solution (5 μg/ml) or TMRE probe at 37°C for 20 min and rinsed twice with PBS. The mitochondrial membrane potential was monitored using a fluorescence microscope to measure the relative fluorescence intensity from mitochondrial JC‐1 monomers or aggregates under 488 and 594 nm laser excitation. In addition, TMRE probe was detected using fluorescence microplate reader (Ex/Em: 550/575 nm).

### 4.12 Measurement of NADPH/NADP ^+^ ratio and total antioxidant capacity

#### 4.12.1 NADPH/NADP ^+^ ratio

The ratio of NADPH/NADP^+^ was measured using a NADP^+^/NADPH Assay Kit with WST‐8 (Beyotime Biotechnology). Cells in six‐well plates after indicated treatment were lysed with NADP^+^/NADPH extract. Lysates were centrifuged at 12,000 *g* for 5 min. Lysates were then separated into two portions. One portion was heated at 60°C to decompose NADP^+^ while the other portion was left on ice as unheated sample. The samples were incubated at 37°C for 20 min in the dark, and then measured at 450 nm using a microplate reader. Protein concentration of each sample was quantified using a BCA protein assay kit. The relative NADPH/NADP^+^ ratio was further standardized by the protein concentration.

#### 4.12.2 Total antioxidant capacity

The total antioxidant capacity was performed with a Total Antioxidant Capacity Assay Kit with FRAP (Beyotime Biotechnology). Cells in six‐well plates after indicated treatment were collected into PBS, sonicated to fully release the antioxidants, and centrifuged at 12,000 *g* for 5 min. The supernatant was collected for detection. FRAP working solution was added to the supernatant and incubated at 37°C for 5 min and then measured at 593 nm using a microplate reader. The total antioxidant capacity was further standardized by the protein concentration.

### 4.13 Immunofluorescent staining

The fibroblast cells cultured in 12‐well plates were fixed with paraformaldehyde for 15 min after indicated treatment; then, the cells were soaked with Triton X‐100 for 10 min. Added 100 μl goat serum to each well and placed at 37°C for 1 h. After aspirating the serum, added 100 μl primary antibody solution and incubated overnight at 4°C. Added fluorescent secondary antibody and incubated for 1 h at room temperature, then stained the nucleus with DAPI for 5 min, added anti‐fluorescence quencher to slow down fluorescence quenching. Wash the cells with PBS three times between each step. Capture the images and use Image‐J software to measure the fluorescence intensity.

### 4.14 Animal experiments

Male C57BL/6J mice at the age of 8‐week‐old were purchased from Ensiweier Biotechnology Co., Ltd. and were raised in the Experiment Animal Center at Chongqing Medical University, P. R. China, according to the National Institutes of Health Guidelines for the Care and Use of Laboratory Animals (NIH Publication No. 85‐23). The mice were kept with constant temperature (22 ± 2°C), 60% relative humidity, 12‐h bright/12‐h dark cycle lighting, and noise below 60 decibels. Mice can freely take in sufficient water and food. The mice were randomly divided into two groups according to whether they were administrated with saline (CON + V group) or D‐gal solution (200 mg/kg body weight, dissolving in 0.9% saline, D‐gal group). After 8 weeks of administration, the mice in D‐gal group were further divided into D + V group, D + LDI and D + HDI group randomly according to whether different concentrations of selective LOXL2 inhibitor treatment were given (D + V: 200 mg/kg D‐gal; D + LDI: 200 mg/kg D‐gal and 5 mg/kg LOXL2 inhibitor; D + HDI: 200 mg/kg D‐gal and 10 mg/kg LOXL2 inhibitor). The mice were intraperitoneally injected once a day for 8 weeks. Body weight and grip strength were recorded every other week. Grip strength of each mouse was recorded using an electronic grip strength meter. Twenty‐four hours after the last injection, all mice were anesthetized by pentobarbital sodium (80 mg/kg), and muscle mass of each mouse was measured using DEXA.

### 4.15 Tissue processing

Mice were weighed and then anesthetized by intraperitoneally injecting pentobarbital sodium (80 mg/kg). Bilateral gastrocnemius muscles were dissected and collected. One muscle was quickly frozen in liquid nitrogen then stored at −80°C, and the other was subjected to 4% paraformaldehyde fixing (4°C overnight), paraffin embedding, and sectioning at a thickness of 5 μm sequentially.

### 4.16 Assessment of skeletal muscle oxidative stress markers

The samples of skeletal muscle were homogenized in certain volume (10% w/v) of ice‐chilled 50 mM phosphate buffer (pH 7.4). After centrifugation at 1000 *g* at 4°C for 10 min, the supernatants were collected for biochemical analysis. The protein concentration was measured by BCA as the standard reference. The activities of SOD and GSH‐PX, and the contents of MDA and GSH in skeletal muscle were determined using corresponding assay kit (Nanjing jiancheng Bioengineering Institute) according to the instructions of manufacturer.

### 4.17 Hematoxylin and eosin (H&E) staining

After sections were dewaxed and hydrated using a dimethylbenzene solution and gradient ethanol, they were stained in hematoxylin for 3 min, then subjected to differentiation for 5 s with 1% hydrochloric acid ethanol to turn the nucleus blue, and then lastly soaked in eosin for 2 min to stain the cytoplasm red. After dehydrated with gradient ethanol and cleared in dimethylbenzene solution, the slides were mounted with neutral resin. Observe the sections under a microscope, and capture the images with a digital camera. Determine the average CSA of gastrocnemius muscle in each specimen by using Image‐J software.

### 4.18 Sirius red staining

All the sections were dewaxed and hydrated, soaked in Sirius Red dye solution for 1 h, rinsed with running water for 30 s, dehydrated with gradient ethanol, cleared in dimethylbenzene solution, and mounted with neutral resin. Images were captured, and collagen volume fraction was calculated using Image‐J software.

### 4.19 Immunohistochemical staining

After sections were dewaxed and hydrated, antigens were restored using citric acid buffer, then treated with 3% hydrogen peroxide for 10 min to inactive endogenous peroxidases, and 10% bovine serum albumin for 30 min to block non‐specific binding. Next, the sections were incubated with primary antibodies at 4°C overnight. Subsequently, the sections were incubated with diluted biotin labeled secondary antibody at room temperature for 30 min and then with diluted streptavidin‐horseradish peroxidase at 37°C for 15 min. Sections were washed with PBS for three times between each of the steps. Lastly, the sections were soaked in 3,3′‐diaminobenzidine (DAB) solution for 5 min, hematoxylin for 3 min, and washed in running water for 30 s. All sections were subjected to differentiation in 1% hydrochloric acid ethanol for 5 s, dehydrated with gradient ethanol, cleared in dimethylbenzene solution, and mounted with neutral resin. Images were captured with a digital camera and analyzed using Image‐J software.

### 4.20 Transmission electron microscope

The partial samples of skeletal muscles were harvested at the dimension of 1 × 1 mm, fixed with 2.5% glutaraldehyde, stained with osmium tetroxide and uranyl acetate, sectioned at the thickness of approximately 50 nm, and then examined under a transmission electron microscope at Institute of Life Sciences, Chongqing Medical University.

### 4.21 Statistical analysis

All data were analyzed and image presented by SPSS and GraphPad Prism software. Comparisons between two groups using Student's *t*‐test, whereas multiple comparisons were analyzed with one‐way ANOVA (Dunnett *t*‐test or Tukey test). *p* < 0.05 was considered as statistically significant. Data are represented as mean ± SD or SEM.

## AUTHOR CONTRIBUTIONS

Y.W, Q.X, J.C, Y.Z, and Y.S designed experiments. Y.W, Y.Y, and Y.W designed and performed experiments. Y.W and Q.X wrote the manuscript. J.Y, J.W, A.G, and Z.L performed experiments. All authors commented, edited, and approved the manuscript.

## CONFLICT OF INTEREST

The authors declare no conflict of interest.

## Data Availability

The data that support the findings of this study are available from the corresponding author upon reasonable request.
